# From Clicks to Care: Enhancing Clinical Decision Making Through Structured Electronic Health Records Navigation Training

**DOI:** 10.3390/jcm14144813

**Published:** 2025-07-08

**Authors:** Savita Ramkumar, Isaa Khan, See Chai Carol Chan, Waseem Jerjes, Azeem Majeed

**Affiliations:** Department of Primary Care and Public Health, Faculty of Medicine, Imperial College London, 86 Wood Lane, White City Campus, London W12 0BZ, UK; savita.ramkumar22@imperial.ac.uk (S.R.); isaa.khan22@imperial.ac.uk (I.K.); carol.chan6@nhs.net (S.C.C.C.); a.majeed@imperial.ac.uk (A.M.)

**Keywords:** electronic health records, medical education, clinical decision making, information retrieval, simulation-based learning, diagnostic reasoning, EHR navigation training

## Abstract

**Background**: The effective use of electronic health records (EHRs) is an essential clinical skill, but medical schools have traditionally provided limited systematic teaching on the topic. Inefficient use of EHRs results in delays in diagnosis, fragmented care, and clinician burnout. This study investigates the impact on medical students’ confidence, efficiency, and proficiency in extracting clinically pertinent information from patient records following an organised EHR teaching programme. **Methods**: This observational cohort involved 60 final-year medical students from three London medical schools. Participants received a structured three-phase intervention involving an introductory workshop, case-based hands-on practice, and guided reflection on EHR navigation habits. Pre- and post-intervention testing involved mixed-method surveys, simulated case tasks, and faculty-assessed data retrieval exercises to measure changes in students’ confidence, efficiency, and ability to synthesise patient information. Quantitative data were analysed using paired *t*-tests, while qualitative reflections were theme-analysed to identify shifts in clinical reasoning. **Results**: All 60 students successfully finished the intervention and assessments. Pre-intervention, only 28% students reported feeling confident in using EHRs effectively, with a confidence rating of 3.0. Post-intervention, 87% reported confidence with a rating of 4.5 (*p* < 0.01). Efficiency in the recovery of critical patient information improved from 3.2 to 4.6 (*p* < 0.01). Students also demonstrated enhanced awareness regarding system-related issues, such as information overload and fragmented documentation, and provided recommendations on enhancing data synthesis for clinical decision making. **Conclusions**: This study emphasises the value of structured EHR instruction in enhancing the confidence and proficiency of medical students in using electronic records. The integration of structured EHR education to medical curricula can better prepare future physicians in managing information overload, improve diagnostic accuracy, and enhance the quality of patient care. Future research should explore the long-term impact of structured EHR training on clinical performance, diagnostic accuracy, and patient outcomes during real-world clinical placements and postgraduate training.

## 1. Introduction

Electronic health records (EHRs) have become integral to modern healthcare by consolidating patient information, improving accessibility, and enabling coordination across clinical teams [1]. Their effective use supports documentation, communication, and data-informed decision making [2]. However, despite their widespread adoption, many healthcare professionals find EHR systems challenging to use efficiently [3], demonstrating the need to embed EHR proficiency into formal medical education [4].

Using EHRs inefficiently can lead to missed information, delays in clinical decision making, and reduced face-to-face patient interaction [5]. For medical students, EHR navigation difficulties may hinder clinical reasoning and the synthesis of patient data [6,7]. As reliance on digital records grows, equipping students with effective EHR skills is increasingly essential.

While medical school equips students with diagnostic and therapeutic skills, it has lagged behind in the implementation of structured EHR teaching [8]. Medical students often acquire EHR skills independently through observation or trial and error, leading to inconsistent proficiency and underuse of essential system functions. This lack of structured teaching jeopardises students’ educational progress and may compromise the quality of the care they provide as future doctors [9,10].

This study aims to evaluate how medical students use EHRs to retrieve clinically significant patient information, and to assess the impact that structured EHR training has on their efficiency and decision making during their placement.

## 2. Methods

### 2.1. Study Design

This cohort study assessed the impact of structured EHR navigation training on medical students’ confidence, efficiency, and ability to retrieve clinically relevant patient information. A pre- and post-intervention format was used to evaluate changes in students’ EHR proficiency following a structured training programme. The intervention consisted of three phases: an introductory workshop, case-based hands-on training, and reflective analysis of EHR usage patterns. Quantitative survey responses and qualitative reflections were analysed to assess the effectiveness of the training. The observational design enabled standardised delivery of training while allowing for the measurement of individual improvement.

A control group was not included in the design, as the intervention was delivered as part of a compulsory teaching session for all final-year students to ensure equitable access within the time-limited clinical rotation schedule.

### 2.2. Intervention

The structured three-phase intervention consisted of (1) an introductory workshop (60 min), (2) case-based hands-on simulation (90 min), and (3) guided reflection and feedback (30 min), (Table 1, Appendix A).

The introductory workshop was delivered by two experienced clinical educators, both of whom are general practitioners with over five years of experience teaching EHR-related content to undergraduate medical students. The session covered core navigation principles, common pitfalls, and best practices, supplemented by live demonstrations in a simulated EHR environment.

The hands-on simulation phase included three clinical cases of graded complexity (simple, intermediate, complex). Each case was built using anonymised, de-identified data from synthetic patient scenarios designed to mimic real-life documentation formats. Tasks ranged from straightforward lab result retrieval to complex medication reconciliation involving multidisciplinary notes and longitudinal trend identification. Students worked independently to complete the cases while faculty observers recorded performance metrics using standardised rubrics.

The final phase was a 30 min faculty-led debriefing in small groups (8–10 students), during which students reflected on their navigation strategies, discussed the challenges encountered, and proposed improvements to their EHR approach. Reflection prompts were based on Kolb’s experiential learning model and aimed to support metacognitive insight in their decision-making processes.

The intervention was completed in a single half-day block integrated into the students’ clinical schedule. Faculty facilitators maintained consistency across groups by following a standardised teaching script and using calibrated rubrics for performance evaluation. This structured approach ensured fidelity and comparability across different student cohorts.

### 2.3. Setting

The intervention was delivered within a structured educational environment using simulated EHR systems replicating real-world patient records under faculty supervision. The EHR system used mirrored platforms common in UK healthcare settings to ensure practical application of skills. Sessions were integrated into students’ clinical schedules to minimise disruptions. Over six weeks, all participants completed the training and assessments.

### 2.4. Participants

Sixty final-year medical students participated. Inclusion criteria required completion of clinical rotations in general medicine, general surgery, and primary care to ensure familiarity with electronic patient records. Recruitment was conducted through announcements during clinical training sessions. Participation was voluntary, and all students provided written informed consent. No exclusions were made based on prior EHR experience to ensure a diverse range of skill levels. All 60 students completed both pre- and post-intervention assessments, achieving a 100% retention rate.

### 2.5. Variables

The study evaluated multiple quantitative and qualitative variables to assess the impact of structured EHR training on students’ ability to navigate electronic records efficiently.

Primary outcome variables included self-reported confidence in EHR use on a five-point Likert scale, with pre- and post-intervention surveys capturing changes in self-assessment. Efficiency of data retrieval was assessed through standardised case scenarios to measure the time of completion and accuracy of data retrieval. The ability to synthesise information was evaluated through structured case analysis, assessing the recognition of red flags, key clinical details, and trends in patient data.

Secondary outcome variables included awareness of systemic barriers to EHR use, measured through self-reported perceptions of information overload, fragmented documentation, and workflow inefficiencies. Common EHR navigation pitfalls were identified through case-based exercises where students documented specific challenges. Written reflections provided further qualitative insights into perceived improvements and ongoing challenges.

Pre- and post-intervention scores were summarised using mean values with standard deviations, with changes assessed for statistical significance. The combination of quantitative survey data and qualitative reflections allowed for a comprehensive evaluation of structured EHR training’s influence on clinical data management.

### 2.6. Data Sources and Measurement

Data collection included pre- and post-intervention surveys, standardised case-based assessments, and reflective journal entries. Surveys measured confidence in EHR navigation, efficiency in retrieving patient information, and awareness of systemic challenges in data management. Confidence and efficiency were rated on a five-point Likert scale, while open-ended questions captured perceived challenges and improvements following training.

Case-based assessment evaluated students’ ability to retrieve and synthesise clinically relevant information within an EHR system. Simulated patient cases included structured clinical data such as consultation notes, laboratory results, and imaging reports. Retrieval performance was assessed based on time to completion, which was measured using digital timers embedded in the simulation platform and confirmed by faculty observers. The accuracy of the extracted data was assessed using a structured rubric measuring the recognition of red flags, clinical trends, and synthesis of key details.

Qualitative data were obtained from students’ reflective journal entries and were analysed thematically using an inductive approach. Two independent researchers first reviewed the data independently and generated initial codes. A shared coding framework was then developed collaboratively through iterative discussion. The coded data were re-analysed using this framework, and emergent themes were compared across researchers. Intercoder agreement was assessed informally through discussion and was strengthened by repeated review cycles. Any discrepancies were resolved by consensus to ensure consistency and analytic integrity. This approach was chosen to enhance the trustworthiness of the analysis and mitigate individual coder bias. Faculty evaluators, meanwhile, assessed performance in the simulation using standardised rubrics.

To validate the assessment of ‘efficiency,’ task time thresholds were established through pilot testing involving three clinical educators who independently completed the simulation tasks. Their average completion times were used as a reference range, and ±2 standard deviations from this mean were applied to define acceptable efficiency performance bands. For ‘accuracy,’ task-specific checklists were developed based on predefined critical data elements (e.g., key lab trends, medication discrepancies, flagged red flags). Accuracy was rated by trained faculty using a structured rubric. Performance measures were piloted and refined in advance of the study to ensure clarity and consistency.

### 2.7. Bias Control

Potential sources of bias were addressed to maintain study validity. Selection bias was minimised by recruiting students from three medical schools to ensure diverse clinical experiences. Although participation was voluntary, all eligible students were invited to reduce self-selection bias towards students who were more confident in EHR navigation.

Response bias was mitigated by including standardised case-based assessments alongside self-reported surveys, ensuring objective measures of EHR navigation efficiency. Observer bias was controlled using structured performance rubrics to assess case-based evaluations. Faculty evaluators were blinded to students’ pre- or post-intervention status. With qualitative data, thematic analysis of reflective journals was independently conducted by two researchers, with discrepancies resolved through discussion. Recall bias was minimised by administering post-intervention assessments immediately after training. The study’s mixed-methods approach balanced subjective self-assessments with objective performance data, strengthening the robustness of findings.

### 2.8. Study Size

Sample size determination was based on an a priori power analysis using G*Power 3.1 software. We selected a two-tailed paired *t*-test design to compare pre- and post-intervention scores. A medium effect size (Cohen’s d = 0.5) was assumed based on comparable educational interventions reported in the literature. The significance level (alpha) was set at 0.05, with a statistical power (1–β) of 0.80, representing an 80% probability of detecting a true effect. Under these parameters, the required minimum sample size was calculated to be 52 participants. To mitigate the risk of attrition and ensure full statistical power, we recruited 60 participants. As all enrolled students completed the intervention and assessments, the final sample size preserved the planned statistical sensitivity to detect meaningful differences.

### 2.9. Quantitative Variables

The study assessed confidence in EHR navigation through self-reported ratings on a five-point Likert scale. Efficiency in retrieving patient data was measured by time taken to locate key clinical information in standardised cases. The accuracy of data extraction was assessed through structured case-based evaluations, where students retrieved past medical history, laboratory results, and imaging reports. Performance was rated using a five-point scale, with higher scores reflecting greater accuracy.

The recognition of systemic barriers in EHR use was measured through survey responses, assessing awareness of information overload and documentation inefficiencies. Improvements in diagnostic reasoning using EHRs were determined by comparing pre- and post-intervention scores across all measured domains. Paired *t*-tests analysed statistical significance, with results reported as mean values with standard deviations.

### 2.10. Statistical Methods

Descriptive and inferential statistical methods were used to assess the impact of structured EHR training. Prior to performing *t*-tests, we assessed the normality of continuous variables using the Shapiro–Wilk test; all data met the assumption for parametric testing (*p* > 0.05). Pre- and post-intervention comparisons of continuous variables, including confidence scores, efficiency in data retrieval, and accuracy, were analysed using paired *t*-tests. A *p*-value of <0.05 was considered statistically significant.

Subgroup analyses were conducted to explore performance differences by gender (male vs. female) and prior EHR exposure (yes vs. no), based on self-reported baseline questionnaires. Independent-samples *t*-tests were used to compare post-intervention accuracy and efficiency scores across these subgroups. A significance level of *p* < 0.05 was used for all tests.

Categorical data, such as the proportions of students reporting high confidence in EHR navigation before and after the intervention, were analysed using McNemar’s test. Effect sizes were calculated using Cohen’s d to measure the magnitude of improvement. Qualitative data from reflective journals were thematically analysed by two independent researchers, with discrepancies resolved through discussion. Statistical analyses were conducted using SPSS version 28.0 (IBM Corporation, Armonk, NY, USA) to ensure robust methodological rigour. Cohen’s d was calculated to report effect sizes for pre- and post-intervention comparisons.

### 2.11. Ethical Considerations

This study was classified as a quality improvement/service evaluation initiative, and formal ethical approval was not required. All participants provided written informed consent, ensuring voluntary participation. Data were collected and analysed anonymously, adhering to institutional data protection guidelines. The findings will contribute to improving medical education by integrating structured EHR training into clinical curricula to enhance diagnostic reasoning and patient safety.

## 3. Results

### 3.1. Participants

A total of 60 final-year medical students participated in the study, all of whom completed the intervention and both pre- and post-intervention assessments, ensuring a 100% retention rate. The participant cohort was diverse in terms of gender, prior clinical exposure, and self-reported familiarity with EHR systems. The group consisted of 34 female students (57%) and 26 male students (43%), with a mean age of 24.1 ± 1.3 years.

All students completed core clinical placements in internal medicine, surgery, and primary care. Additionally, 21 students (35%) had prior experience in a subspecialty rotation, such as Emergency Medicine and Geriatrics, which introduced them to different EHR systems across clinical settings.

The baseline survey data indicated that 38 students (63%) had never received formal training in EHR navigation, while 22 students (37%) reported informal exposure through clinical rotations or peer-led learning. The self-reported confidence levels in using EHRs varied widely, with only 28% of students reporting high confidence in their ability to retrieve key patient data, while 50% reported moderate confidence and 25% reported low confidence (Table 2).

The diverse range of clinical experiences and baseline EHR proficiency levels allowed for a comprehensive evaluation of the effectiveness of structured EHR training in enhancing medical students’ clinical efficiency and data synthesis skills.

### 3.2. Descriptive

Baseline assessments revealed significant variability in students’ confidence and efficiency in EHR navigation (Table 2). Despite this, 84% of participants agreed or strongly agreed that EHR navigation was essential for clinical efficiency and patient safety.

Pre-intervention task simulations showed that students took an average of 8.5 ± 1.6 min to complete standardised EHR tasks, with faster completion times for simple tasks such as retrieving recent laboratory results (6.1 ± 1.2 min) and longer times for complex tasks such as reconciling medication discrepancies (11.3 ± 1.7 min). Students’ accuracy in retrieving key clinical data varied by task complexity, with a 92% accuracy rate in simple retrieval tasks but a 71% accuracy rate in multi-step tasks requiring data synthesis.

Subgroup analysis showed no statistically significant differences in mean accuracy scores between male and female students (Mean ± SD: 84.2% ± 8.1 vs. 83.5% ± 9.0, *p* = 0.68) or between those with and without prior EHR exposure (85.0% ± 7.5 vs. 83.0% ± 9.6, *p* = 0.29). Similarly, efficiency scores (measured as time to task completion) did not differ significantly by gender (12.4 ± 2.1 min vs. 12.6 ± 2.4 min, *p* = 0.57) or prior EHR experience (12.2 ± 2.0 min vs. 12.8 ± 2.3 min, *p* = 0.33). These results suggest broadly similar gains across subgroups.

Pre-study reflections highlighted several recurring challenges in EHR navigation, including difficulty filtering large amounts of information, locating older patient records, and synthesising data from multiple sources. A common theme was the perception that navigating EHRs was inefficient and time-consuming, with one participant stating, “It feels like searching for a needle in a haystack when trying to find specific patient notes.” (Table 3).

The pre-intervention findings established a clear need for structured training to enhance students’ ability to extract, interpret, and apply patient information efficiently.

### 3.3. Outcome Data

The post-intervention results demonstrated statistically significant improvements in multiple key domains.

**Figure 1:**
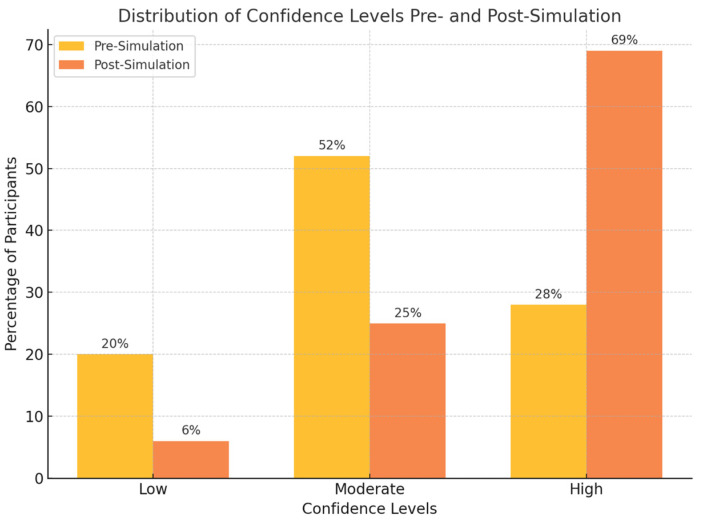
Stacked bar chart visualising the distribution of confidence levels pre- and post-simulation. Pre-intervention, 28% of students reported high confidence, 52% moderate, and 20% low. Post-intervention, 69% reported high confidence, 25% moderate, and 6% low. The chart highlights the significant increase in “High” confidence after the simulation and the reduction in “Low” and “Moderate” confidence levels.

Confidence in EHR navigation increased substantially, with 69% of students reporting high confidence after training, compared to 28% pre-intervention (*p* < 0.01, Cohen’s d = 1.02, 95% CI: 0.76 to 1.28). This change corresponds to a large effect size (Cohen’s d = 1.02, 95% CI: 0.76 to 1.28). The percentage of students strongly agreeing that EHR navigation is integral to clinical efficiency and patient safety increased from 84% to 96% (Table 2, Figure 1).

**Figure 2:**
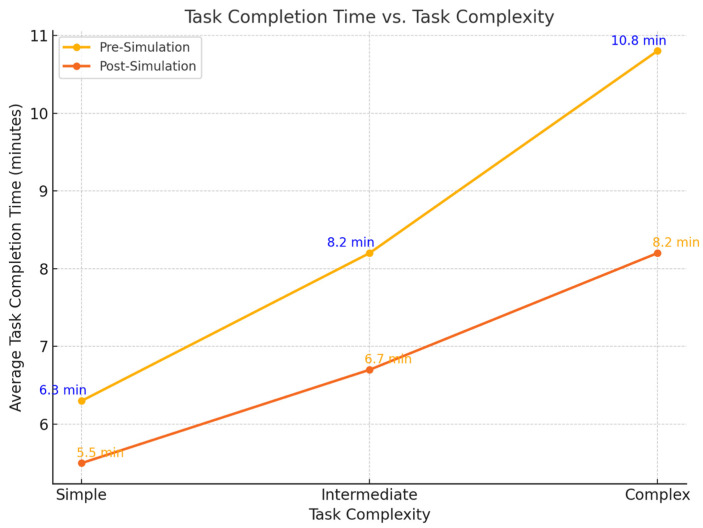
Line graph illustrating the average task completion time for different task complexity levels (simple, intermediate, complex) before and after the simulation. It demonstrates the improvement in efficiency across all complexity levels after the simulation training.

Students’ efficiency in retrieving patient data improved significantly, with the average time to task completion decreasing from 8.5 ± 1.6 min pre-intervention to 6.9 ± 1.2 min post-intervention (*p* < 0.01, Cohen’s d = 1.10, 95% CI: 0.84 to 1.36) (Figure 2). Performance on simple tasks improved marginally, while efficiency in multi-step tasks (e.g., medication reconciliation and identifying longitudinal data trends) showed the largest improvement, with completion time reduced by 23%.

Accuracy rates also improved, particularly in complex cases requiring data synthesis. While pre-intervention accuracy for retrieving simple patient data was 92%, students initially struggled with tasks requiring cross-referencing multiple data points, achieving only 71% accuracy. Post-intervention, accuracy in multi-step tasks increased to 82%, reflecting an enhanced ability to integrate information from different EHR sections and synthesise clinically relevant details. The improvement in accuracy (from 71% to 85%) yielded a medium-to-large effect size (Cohen’s d = 0.78, 95% CI: 0.52 to 1.04).

Students’ reflections highlighted a greater appreciation for the complexity of EHR systems and the cognitive strategies required for efficient navigation. The structured debriefing sessions revealed that many students had underestimated the time, effort, and attention to detail required to extract meaningful patient data. One participant noted, “This exercise completely changed my perception of EHR navigation. I realised it’s not just about speed but knowing where to look and how to filter information effectively.”

Students also reported an increased ability to recognise systemic inefficiencies within EHRs, including cluttered documentation, inconsistent formatting, and difficulty in synthesising data across specialties. One common concern was that excessive, disorganised information often led to cognitive overload, making it harder to distinguish critical patient details from redundant entries.

Students who used advanced EHR functions such as filters, search features, and summary views significantly outperformed those who relied on manual scrolling. Participants who actively utilised filters completed tasks 24% faster than their peers, yet only 43% of students reported confidence using advanced system tools before the intervention, despite these tools being linked to faster task completion. After the intervention, 68% of students reported confidence in using these tools, reflecting a notable gain in digital literacy.

### 3.4. Other Analyses

Further subgroup analyses showed that students with prior exposure to EHRs in primary care settings performed significantly better in tasks requiring cross-disciplinary data synthesis, such as reconciling multi-specialty notes or identifying key red flags in patient records. This suggests that experience in settings where continuity of care is emphasised may enhance EHR navigation skills. Qualitative thematic analysis of students’ post-intervention reflections identified three dominant themes:

1. Recognition of the role of EHR navigation in clinical decision making—Many students expressed a new awareness that poor EHR efficiency could lead to delayed diagnoses, fragmented care, or missed critical findings.
2. Appreciation of cognitive strategies for effective data retrieval—Students reported improved confidence in structuring their approach to retrieving clinical information, rather than relying on a disorganized or trial-and-error method.
3. Challenges with system-related inefficiencies—Many students highlighted structural problems within EHR design, such as poor search functionality, inconsistent formatting of notes, and excessive redundant data, which all contributed to cognitive burden when making clinical decisions.

Students also proposed actionable recommendations for improving EHR training, including

**Figure 3:**
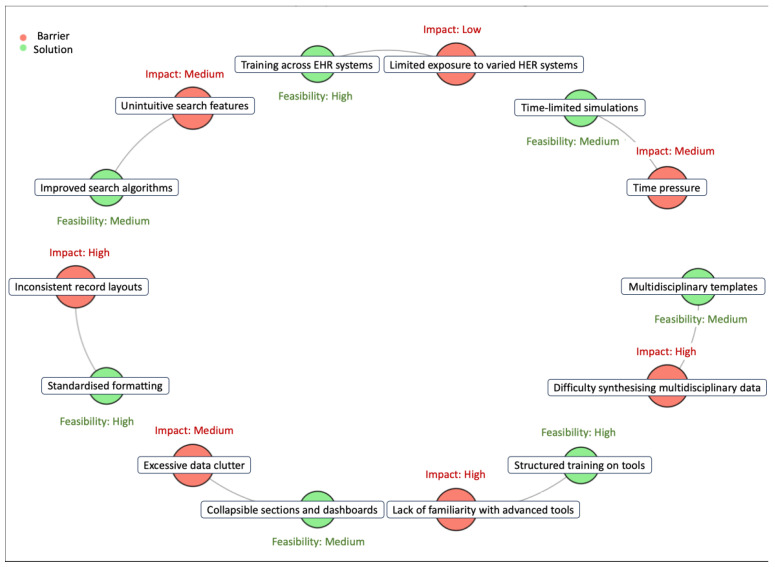
Refined concept map: barriers and solutions in EHR navigation. This concept map visualises the key barriers faced by medical students during EHR navigation and their corresponding proposed solutions. Barriers, such as inconsistent record layouts and excessive data clutter, are highlighted for their high impact on task efficiency and accuracy. Solutions, like standardised formatting and collapsible data sections, are annotated with feasibility ratings to emphasise practical interventions. The directional connections illustrate how each barrier links to actionable remedies, aligning with findings that structured training and systemic improvements are critical for optimising EHR usability. This map demonstrates the importance of targeted strategies in enhancing clinical efficiency and decision making.

1. Enhanced education on advanced system features such as filtering, search shortcuts, and integration of data across different sections.
2. Case-based training modules tailored to common clinical scenarios to provide structured practice in synthesising patient information.
3. Interdisciplinary workshops incorporating perspectives from different healthcare professionals to improve the understanding of how various specialties interact with EHRs (Figure 3).

## 4. Discussion

### 4.1. Key Results

This study highlights the importance of structured EHR teaching during undergraduate medical education. Despite medical students recognising the critical role that EHR navigating has in clinical practice, their baseline proficiency levels are often inadequate to meet the actual needs of the workplace. The pre-test results revealed significant gaps in EHR efficiency and accuracy, particularly in more complex, multi-step procedures to synthesise information. These gaps were largely the result of the inability to retrieve, sift, and synthesise information across different EHR sections, leading to delays and errors in clinical decision making.

After structured simulation-based teaching, students demonstrated significant increases in confidence, efficiency, and accuracy when using EHRs. Confidence in retrieving patient information increased, and task completion times also significantly improved, especially for more complex multiple-step tasks. Accuracy in the extraction of critical clinical information increased, demonstrating the impact that structured EHR teaching has on clinical reasoning and data synthesis.

The study also discovered that students did not sufficiently use the advanced capabilities of EHRs, such as filtering and searching. This presents an area that future training should be geared towards, ensuring that students effectively use system functions to maximise workflow and reduce mental load. The findings affirm the need for structured, simulation-based EHR teaching to address these issues and prepare students with efficient clinical practice skills.

### 4.2. Limitations

While this study has strong supporting evidence regarding the benefit of structured EHR teaching, several limitations must be noted. One limitation is that the simulated EHR environment, as much as it aims to mimic actual systems, may simplify or not capture the complexity of different clinical environments. The variation in the EHR interfaces within different healthcare institutions means that the students may still face the issue of adaptation when using the actual systems in the real-world environment.

In addition, the lack of a control or comparator group limits causal attribution of the observed improvements. This design choice was made to ensure that all final-year students received access to the structured training during their clinical placements, but it does introduce the possibility that other factors—such as increased task familiarity or general educational maturation—may have influenced outcomes.

Another limitation is the sample size, as 60 participants is relatively modest; moreover, participants were geographically restricted to three London medical schools. Although participation was invited universally and the training was embedded in scheduled teaching, the voluntary nature of participation may still introduce self-selection bias. Students more confident or interested in digital systems may have been more inclined to participate fully, potentially inflating reported improvements.

Although we conducted exploratory subgroup analyses by gender and prior EHR exposure, no statistically significant differences were observed. These findings suggest broadly comparable outcomes, but given the modest sample size, the study may have been underpowered to detect smaller subgroup effects.

In addition, although we used standardised rubrics for assessing accuracy and efficiency, we acknowledge the potential for residual subjectivity. Scoring was performed by trained faculty members who underwent a calibration process before the intervention, which involved consensus marking of sample responses to ensure consistent interpretation of rubric criteria. During the study, faculty independently scored a random subset of cases to check for inter-rater reliability. However, some level of interpretive judgement is unavoidable in educational assessment and remains a limitation of our approach.

The findings might not be fully transferable to other institutions, health systems, or countries where different EHR platforms and learning methods are utilised. Moreover, the time constraints that were simulated during task simulations may have artificially inflated error rates, as students had to work on tasks with close deadlines. While the constraints on time were designed to simulate the pressures that are found in actual clinical settings, the constraints may not reflect the typical student learning curves when using EHRs in less pressure-ridden environments.

Additionally, the possibility of a Hawthorne effect must be considered [11], as students may have altered their behaviour or performed better during simulation tasks due to the awareness that their performance was being observed and evaluated.

Finally, self-rated confidence could be subjected to response bias. The students may have overestimated their confidence as a result of perceived expectations rather than mastery of skill in using EHRs. Stronger validation of skill retention and clinical impact would be offered by future work that incorporated objective, long-term measurement in real-world clinical environments.

### 4.3. Interpretation

The findings affirm that EHR navigation remains a significant concern even for medical students who are already proficient with computer interfaces. The students reported having issues with the retrieval and synthesis of critical patient information, particularly when presented with the need to decipher multidisciplinary notes or reconcile medication discrepancies. This corroborates with Wilbanks et al. [12] and Chan et al. [13], who noted the usability hurdles in EHRs that compromise clinical efficiency as well as patient safety.

Students also struggled with the inconsistent record layouts and varying formatting across different parts of the EHRs, causing increased task completion times and error rates. Specifically, in tasks that required integration of specialist notes, lab trends, and medication histories, there is a need for more standardisation in the EHR system design.

The primary barrier that arose during this research was information overload, with students experiencing issues in identifying relevant clinical information when faced with an excessive amount of documentation. This result supported other studies that have indicated that excessive levels of cognitive load result in lower accuracy and increased decision-making errors [14,15]. Contributing to the issue was the underuse of search filters and structured data tools, which could potentially bring about efficiency but were rarely used because the staff had not received adequate training.

The results emphasise the necessity for systematic EHR instruction as part of medical curricula. Despite the recognition by students of the importance of EHR proficiency, students reported limited formal instruction, preferring to learn through informal, observational learning during clinical rotations. This aligns with Wald et al. [16] and Rouf et al. [17], who also observed the absence of systematic EHR teaching in medical schools. Meanwhile, Cuddy et al. [9] found significant variation in EHR teaching across medical schools in the United States’, thus creating uneven preparedness on the part of the entering residents.

As with Rajaram et al. [18], this study also advocates for structured teaching of EHR use to better prepare physicians for the practice setting. The growing consensus in medical school teaching suggests that EHR teaching must be integrated as a core competency rather than an ad hoc observation skill.

Simulation instruction, as demonstrated in this study, offers an effective model for structured learning by combining technical competence with practical problem-solving. The increasing application of simulation in medical school has been supported by Elendu et al. [19], who emphasised its value in educating future healthcare professionals. Similarly, the use of a 90 min cognitive EHR training session by Zavodnick et al. [20] demonstrated that structured, hands-on teaching has a significant effect on student competence.

Aside from medical school, structured EHR training has resulted in improvements in patient safety and clinical efficiency. A study by Tubaishat [21] showed that EHR systems reduce medication errors, enhance documentation accuracy, and improve continuity of care.

### 4.4. Future Directions

These findings pave the way for additional work on the long-term retention of skills and the clinical application of organised EHR instruction. Longitudinal studies following students through resident training would be enlightening regarding the long-term development of EHR proficiency. Studies by Adler-Milstein et al. [22] demonstrated long-term improvement through EHR implementation, suggesting that organized instruction may lead to long-term benefits to clinical efficiency and patient outcomes.

The expansion of EHR training across different platforms would also enhance preparedness, as medical graduates often alternate through different EHR systems when practicing. However, Kruse et al. [23] reported that implementation of EHR training faced barriers, including budget limitations, technical problems, and resistance to change. However, low-cost models, for instance the simulation model piloted by Cristiano et al. [24], provide feasible solutions for integrating EHR training into the current curricula.

The future direction of EHR usability might also be impacted by innovations in AI, including predictive analytics, cognitive searching, and clinical decision support based on machine learning. Rose et al. [25] examined the use of AI in EHR systems, highlighting the benefits as well as the ethical considerations involved in using AI to reduce the burden on doctors and improve data interpretation. As AI continues to develop, its role in EHR navigation and usability will likely be even more important, requiring new approaches to teach medical students about AI-based clinical workflows.

While this study demonstrated measurable improvements in simulated task performance, it did not assess students’ actual clinical behaviours or outcomes during real-world placements. These findings should therefore be interpreted as evidence of improved preparedness rather than direct evidence of enhanced clinical performance. Future, longitudinal research should examine whether gains in simulated EHR navigation translate into sustained improvements in clinical settings, including diagnostic accuracy, workflow efficiency, and patient safety. Ideally, this would involve tracking students through clinical rotations and postgraduate training to determine how effectively they apply these navigation skills in real-world patient care.

### 4.5. Generalisability

While the research findings are informative about EHR instruction in medical students, the sample size and geographic setting restrict the generalisability. Hence, the findings may not be fully transferable to different healthcare systems, curricula, or populations. Studies with larger, more heterogeneous cohorts across different regional and specialty settings are needed to verify these findings. Despite these limitations, the underlying concepts discovered in this work, such as the value of structured EHR instruction, the benefit of simulation learning, and the value of synthesis skills with data, are broadly transferable to all medical educational settings globally. Further research must look at multi-institutional rollouts to assess whether the same increases in confidence, efficiency, and accuracy are seen in more heterogeneous, larger populations.

## 5. Conclusions

This study emphasises the need for structured EHR training in preparing medical students for efficient, accurate, and safe clinical practice. Integrating simulation-based learning in medical curricula has the potential to prepare future physicians better to deal with complex computer systems and enhance diagnostic accuracy, clinical decision making, and patient care outcomes.

## Figures and Tables

**Table 1:** Phased assessment framework for evaluating EHR navigation skills.

Phase	Key Activities	Objectives	Metrics Assessed
Pre-Assessment	- Participants completed a baseline survey assessing confidence in EHR navigation and its relevance to clinical efficiency. - Open-ended questions gathered insights into previous EHR experiences and perceived challenges.	- Evaluate baseline proficiency and attitudes toward EHR navigation. - Identify gaps in prior training or experiences.	- Confidence levels in navigating EHRs (self-reported). - Perceived importance of EHR navigation in patient care.
Task Simulation	- Students completed standardized scenarios requiring retrieval of key patient data (e.g., labs, notes, medication records) in a simulated EHR environment. - Tasks mirrored real-world challenges like data reconciliation and trend analysis.	- Assess practical EHR navigation skills in locating and interpreting patient data. - Identify common barriers to efficiency and accuracy.	- Time taken to complete tasks (efficiency). - Correct retrieval and interpretation of patient data (accuracy). - Effective use of system tools (navigation).
Post-Assessment	- Participants reflected on simulation challenges and proposed improvements during debriefing sessions. - A post-survey measured changes in confidence and awareness of EHR navigation’s role in patient care.	- Analyse how task performance influences perceptions and attitudes. - Gather actionable feedback for system improvements.	- Change in confidence levels post-simulation. - Qualitative feedback on EHR challenges and learning outcomes.

**Table 2:** Summary of key performance metrics before and after EHR simulation training.

Metric	Baseline	Post-Simulation	Change	Key Observations
Confidence in locating patient data	28%	69%	+41%	Confidence levels improved significantly, with most students attributing the increase to hands-on practice.
Perceived importance of EHR navigation	81%	93%	+12%	Students increasingly recognised EHR navigation as a critical clinical skill after the simulation.
Average task completion time (minutes)	8.2 ± 1.4 min/task	6.7 ± 1.1 min/task	−1.5 min/task	Students became faster at retrieving key data, particularly in straightforward tasks like lab results.
Accuracy in retrieving patient data (complex tasks)	71%	85%	+14%	Accuracy improved most in tasks requiring data reconciliation, reflecting better system navigation skills.
Percentage of students confident using advanced EHR tools	43%	68%	+25%	Use of filters and search functions increased significantly, highlighting the value of targeted training.
Students reporting improved EHR understanding	N/A	90%	N/A	The majority of students reported greater understanding of the importance of EHR systems post-simulation.
Awareness of systemic EHR barriers	64%	88%	+24%	Students became more aware of structural inefficiencies within EHR systems, including data overload.
Students feeling prepared for future EHR use	34%	78%	+44%	Marked improvement in students’ perception of their readiness to use EHRs in clinical practice.

**Table 3:** Identified barriers in EHR navigation and proposed solutions based on participant feedback.

Barrier Identified	Impact on Performance	Proposed Solutions	Student Feedback Examples
Inconsistent record layouts	Increased task completion time due to difficulty navigating sections with varying formats and headings.	Standardised formatting of EHR sections across departments to streamline navigation and reduce confusion.	“It took longer to find results in some sections because the layout wasn’t intuitive.”
Excessive data clutter	Students struggled to identify relevant information amidst irrelevant or outdated entries.	Implement collapsible sections and data summary dashboards to highlight critical patient information.	“There’s so much information that it’s hard to know what’s relevant without going through everything.”
Lack of familiarity with advanced tools	Inefficient navigation resulted in slower task completion and higher error rates in complex scenarios.	Provide structured training on EHR tools like filters, shortcuts, and search functions in medical school.	“I didn’t even realise there were filters available to help narrow down the data.”
Difficulty synthesising multidisciplinary data	Errors in interpreting clinical relevance due to fragmented notes from multiple specialties.	Develop multidisciplinary templates and better-integrated documentation formats for clearer synthesis.	“Reading through separate notes from different teams made it hard to get a complete picture.”
Time pressure during navigation	Stress led to reduced accuracy, particularly in tasks requiring detailed searches under timed conditions.	Incorporate time-limited simulation exercises to build resilience and proficiency under pressure.	“The time limit made me panic and miss some details that I would normally catch.”
Limited exposure to varied EHR systems	Students found the system unfamiliar, especially those who had used a different her systems during rotations.	Expose students to multiple EHR systems through standardised training modules or virtual simulations.	“It was hard to adjust because the system is different from the one I used on placement.”
Unintuitive search features	Students wasted time navigating non-specific search results, leading to frustration and inefficiency.	Improve search algorithms and include predictive text or common query suggestions for faster retrieval.	“The search function wasn’t as smart as I thought, which slowed me down a lot.”

## Data Availability

The datasets generated and/or analysed during the current quality improvement project are not publicly available due ethical reasons but are available from the corresponding author (W.J.) upon reasonable request.

## Appendix A

**Pre-Assessment Survey**

**Objective**: To assess baseline proficiency, confidence, and attitudes toward EHR navigation before the simulation.

**Instructions**: Please respond to the following questions. For statements, select the option that best represents your current level of agreement.

**Demographic Information**:

1. Age: _______
2. Gender: _______
3. Clinical rotations completed (check all that apply):
  ○ Medicine
  ○ Surgery
  ○ Primary Care
  ○ Other: _______

**Likert Scale Statements** (Rate from 1 = Strongly Disagree to 5 = Strongly Agree):

1. I feel confident in navigating electronic health record (EHR) systems to locate patient data.
2. I understand the importance of EHR navigation for clinical efficiency and patient safety.
3. I can efficiently retrieve specific data, such as lab results, medication histories, or physician notes, from EHR systems.
4. I am familiar with advanced EHR tools, such as filters and search functions.
5. I believe my EHR navigation skills affect my ability to deliver patient-centred care.

**Open-Ended Questions**:

1. Describe your previous experience using EHR systems.
2. What do you find most challenging about navigating EHRs?
3. How do you think EHR navigation impacts clinical decision-making and patient care?

**Post-Assessment Survey**

**Objective**: To evaluate changes in proficiency, confidence, and perceptions of EHR navigation after the simulation.

**Instructions**: Please respond to the following questions. For statements, select the option that best represents your level of agreement after completing the simulation.

**Likert Scale Statements** (Rate from 1 = Strongly Disagree to 5 = Strongly Agree):

1. I feel more confident in navigating EHR systems to locate patient data.
2. I now recognize the importance of EHR navigation for clinical efficiency and patient safety.
3. I can effectively use advanced EHR tools, such as filters and search functions.
4. This simulation has improved my ability to retrieve and synthesize patient data efficiently.
5. I feel more prepared to navigate EHR systems in real-world clinical practice.

**Open-Ended Questions**:

1. What was the most valuable lesson you learned during the EHR simulation?
2. What challenges did you encounter, and how did you overcome them?
3. How do you think this experience will influence your approach to EHR use in the future?
4. What improvements would you suggest for EHR training in medical education?

**Task Simulation Evaluation Form**

**Objective**: To record performance metrics during the EHR simulation tasks.

**Task-Specific Metrics**:

1. Task ID: _______
2. Time Taken to Complete Task: _______ minutes
3. Accuracy of Data Retrieval (Check One):
  ○ Completely Accurate
  ○ Partially Accurate
  ○ Inaccurate

**Navigation Metrics**:

4. Use of Filters (Yes/No): _______
5. Use of Search Functions (Yes/No): _______
6. Efficient Navigation (Subjective Rating):
  ○ 1 = Very Inefficient
  ○ 5 = Very Efficient

**Qualitative Notes**:

7. Challenges encountered during this task: ______________________________________________
8. Strengths in navigation: ___________________________________________________________
9. Observed gaps in proficiency: ______________________________________________________

**Debriefing Session Reflection**

**Objective**: To facilitate reflection on the simulation experience and gather qualitative insights.

**Instructions**: During the debriefing session, respond to the following prompts in discussion or written format.

**Prompts**:

1. Reflect on your overall experience with the EHR simulation. What did you learn?
2. Which task was the most challenging, and why?
3. How did you approach overcoming barriers to navigation?
4. What changes would you suggest for improving EHR systems to make them more user-friendly?
5. How has this experience influenced your understanding of the role of EHR navigation in clinical efficiency and patient safety?
